# Supplementary material for: Large Field Alpha Irradiation Setup for Radiobiological Experiments
Source: Methods Protoc. 2019 Aug 28;2(3):75. doi: 10.3390/mps2030075 (PMC6789741; doi:10.3390/mps2030075)
Supplement: Supplementary file 1 [file Design S1.pdf › Design S1.pdf]

700.0000
30.0000
20.0000
25.0000
100.0000
360.0000
400.0000
25.0000
50.0000
265.0000
30.0000
50.0000
30.0000
